# *Shigella flexneri* 1c bacteremia in a child: a case report

**DOI:** 10.1186/s13099-026-00804-w

**Published:** 2026-02-14

**Authors:** Mehrzad Sadredinamin, Zohreh Ghalavand, Maryam Rostamyan, Raana Kazemzadeh Anari, Seyyed Mohammadreza Hosseini Imeni, Bahram Nikmanesh

**Affiliations:** 1 Department of Microbiology, School of Medicine, Shahid Beheshti University of Medical Sciences, Tehran, Iran; 2 Department of Pediatrics Infectious Diseases, Pediatrics Center of Excellence, Hakim Hospital, Tehran University of Medical Sciences, Tehran, Iran; 3 Student Research Committee, School of Medicine, Shahid Beheshti University of Medical Sciences, Tehran, Iran; 4 Department of Medical Laboratory Sciences, School of Allied Medical Sciences, Tehran University of Medical Sciences, Tehran, Iran; 5 Zoonoses Research Center, Tehran University of Medical Sciences, Tehran, Iran

**Keywords:** *Shigella flexneri*, Serotyping, Bacteremia, Diarrhea, Virulence factors, Children

## Abstract

**Background:**

Shigellosis is a significant public health issue in developing countries, particularly affecting young children under the age of five. While it primarily causes gastrointestinal infections, there are rare complications, such as bacteremia, that mainly occur in young children. We present a case of dysentery caused by *Shigella flexneri* serotype 1c, followed by bacteremia.

**Case presentation:**

A 13-month-old Afghan child presented with fever, dysentery, distended abdomen, mild tenderness, and dehydration. He did not respond to empirical treatment with ceftriaxone. However, he was successfully treated with a combination of gentamicin and ciprofloxacin. This is the first report of a patient in Iran who was positive for *S. flexneri* serotype 1c and harbored the *ipaB*, *ipaC*, *ipaD*, *ipaH*, *ipgD*, *virA*, and *sen* virulence factors.

**Conclusions:**

This case alerts clinicians to consider the possibility of *Shigella* bacteremia in young children. Early and accurate diagnosis can improve management and prognosis to reduce the risk of fatality outcomes. Additionally, it emphasizes the need to characterize the role of *Shigella* spp. virulence genes in causing bacteremia.

## Background

Although the prevalence of Shigellosis has recently decreased, it continues to be an essential public health problem in developing countries [1]. This bacterium is a highly transmissible enteric pathogen associated with the third leading cause of diarrheal deaths among children under five [2]. Four species are responsible for Shigellosis, among which *S. flexneri* and *S. sonnei* are the most common causes in developing countries, including Iran [3–5]. *Shigella* spp. is more often the cause of moderate-to-severe diarrhea (MSD) and dysentery among children aged < 5 years living in developing or under-developed countries [6]. However, extraintestinal complications, including bacteremia, are a relatively rare event, occurring primarily in neonates, malnourished children, and the immunosuppressed [7]. In this article, we present a case of a 13-month-old infant who developed episodes of dysentery caused by *S. flexneri* serotype 1c, which was followed by bacteremia.

## Case presentation

A 13-month-old Afghan boy was admitted to Hakim Hospital in Tehran, Iran, on 17 November 2024, after experiencing multiple episodes of dysentery and a 38 °C fever for the past five days. The child belonged to a low socio-economic status and lived in the suburbs of Tehran. Recently, all of his family members had experienced dysentery.

On physical examination, the infant weighed 10 kg. He was ill-looking, with a distended abdomen, mild tenderness in the periumbilical region, and 6% dehydration. Prior to prescribing antibiotics, blood and stool cultures, along with other relevant investigations, were conducted. Stool cultures were done three times. Three sets of blood culture, each containing 3 mL of blood, were transferred into each blood culture bottle under sterile conditions, and cultures were processed using a manual blood culture system. The blood analysis from the laboratory showed the following results: hemoglobin level was 10.3 g/dl, and the total leukocyte count was 7300/µl (Reference Range: 4500–11000/µl). This included 47% neutrophils, 38% lymphocytes, 12% monocytes, 3% eosinophils, and 0% basophils. The platelet count was 151,000 /µl. Additionally, the serum sodium concentration was 132 mEq/L, the erythrocyte sedimentation rate (ESR) was 52 mm/hr, the BUN level was 7 mg/dL, the creatinine level was 0.3 mg/dL, and the C-reactive protein was measured at 385 mg/L. The stool results indicated many WBCs, many/hpf; RBC, 8–10/hpf; and no parasite found. The child was immediately rehydrated with dextrose saline with potassium chloride supplementation. Intravenous ceftriaxone (500 mg) was administered every 12 h.

To help reduce fever, intravenous acetaminophen (Apotel) 100 mg was prescribed, with a maximum of every six hours. The ultrasound revealed no signs of invagination (intussusception), in which one segment of intestine telescopes inside another in the patient.

However, the patient continued to experience fever and dysentery and did not respond to initial empirical treatment. As a result, ciprofloxacin (100 mg) was replaced with ceftriaxone (500 mg), administered every 12 h. The cultures and susceptibility reports were completed five days after the patient was admitted to the hospital. Based on these results, gentamicin (25 mg) was added to the treatment regimen every 8 h. After 48 h, the frequency of diarrhea gradually decreased. The treatment was continued for 10 days, and the patient was discharged in stable condition after a 14-day hospital stay.

**Fig. 1:**
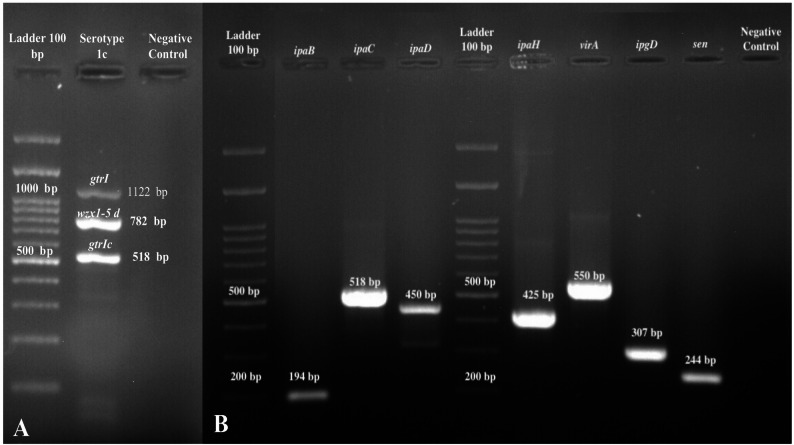
**A: **Multiplex PCR product of *S. flexneri* serotyping. Lane 1, Molecular size markers (100-bp DNA ladder; SMOBIO, South Korea); lane 2, *S. flexneri* serotype 1c; lane 3, Negative control. **B:** PCR products of *S. flexneri* virulence factors genes. Lane 1, *ipaB*; lane 2, *ipaC*; lane 3, *ipaD*, lane 4, Molecular size markers (100‐bp DNA ladder; SMOBIO, South Korea), lanes 5, *ipaH*; lane 6, *virA*; lane 7, *ipgD*; lane 8, *sen*; lane 9, Negative control

As mentioned previously, stool and blood samples were cultured for bacterial identification. The cultures from both blood and stool tested positive for *S. flexneri* in the first two sets. However, the third set of tests for both blood and stool cultures was negative, after which appropriate antibiotics were prescribed. According to CLSI guidelines, the isolate was sensitive to ceftazidime (CAZ; 30 µg), meropenem (MR; 10 µg), doripenem (DOR; 10 µg), ertapenem (ETP; 10 µg), doxycycline (DXT; 30 µg), chloramphenicol (C; 30 µg ), ciprofloxacin (CIP; 5 µg), levofloxacin (LEV; 5 µg), ofloxacin (OFX; 5 µg), gatifloxacin (GAT; 5 µg), minocycline (MN; 30 µg), tigecycline (TGC; 15 µg), azithromycin (ATH; 15 µg), gentamicin (GM; 10 µg), tetracycline (T; 30 µg), susceptible dose dependent to cefepime (FEP; 30 µg), intermediate to imipenem (IPM; 10 µg), amikacin (AK; 30 µg), nalidixic acid (NA; 30 µg) and resistant to ampicillin (AM; 10 µg), cefotaxime (CTX; 30 µg), ceftriaxone (CRO; 30 µg), cefixime (CFM; 5 µg) and cotrimoxazole (SXT; Trimethoprim 1.25 µg, Sulfamethoxazole 23.75 µg) by the Kirby-Bauer method using antibacterial disks (Mast, UK) [r^8^]. The minimum inhibitory concentration (MIC) of azithromycin, determined by the agar dilution method, was 2 µg/ml, indicating susceptibility to the antibiotic. Based on the Multiplex PCR and PCR methods described previously, *S. flexneri* belonged to serotype 1c and also harbored *the ipaB*, *ipaC*, *ipaD*, *ipaH*, *ipgD*, *virA*, and *sen* virulence genes [3, 9] (Fig. 1).

### Discussion and conclusions

*Shigella* infections primarily affect the gastrointestinal system. In contrast, extraintestinal infections, such as those affecting the bloodstream, are rare and mainly occur in children and immunocompromised adults [10, 11]. Specifically, bacteremia is seen in only 0.4% to 7.3% of cases in heterogeneous populations, including children to adults and in patients with cystic fibrosis, and this is mainly linked to infections caused by *S. flexneri* and *S. dysenteriae* [11–14]. *Shigella*-induced bacteremia should be regarded with seriousness because it can significantly increase mortality rates [12, 15, 16]. In this case, the infection was caused by *S. flexneri*, consistent with previous case reports [17–19]. However, some studies indicate that other *Shigella* species can also cause bacteremia [11, 20–22].

Multiplex PCR results indicated that *S. flexneri* serotype 1c, which was detected in the patient, has increased in developing countries over the past decade [23]. The result was inconsistent with previous results done in the adult population, which documented serotypes 1b, 2, 2a, 3, and 4a as the responsible serotypes [24–26]. Notably, these earlier serotypes were isolated from adult populations.

The reasons behind the development of bacteremia after a gastrointestinal infection are not fully understood. However, several factors may contribute, including the virulence of the pathogen, the initial bacterial burden, damaged mucosal barriers, and weakened host immunity [7].

*Shigella* pathogenesis involves several virulence factors encoded on chromosomal pathogenicity islands and a virulence plasmid. The *ipa* and *ipg* genes are crucial in invading epithelial cells and initiating *Shigella* infection [27]. The invasion plasmid antigen H (*ipaH*), a key target for the molecular identification of *Shigella* species, is essential in invading and modulating host inflammatory responses during bacterial infections [28]. We also identified the *ipaB*, *ipaC*, and *ipaD* genes, which encode proteins playing a crucial role in colonic epithelial cell invasion and intracellular survival, as well as translocating various effector proteins [9]. The invasion plasmid gene D (*ipgD)*, which enhances immune evasion and facilitates cell-to-cell spread by disrupting communication between host cells, was identified in our isolate [27, 29]. The *virA* is another virulence factor in our isolate and is involved in *Shigella*’s uptake, invasion, and cell-to-cell transmission within the human host [30]. Our patient continued to have watery diarrhea during the hospitalization, which is hypothesized to be associated with the presence of the *sen* gene [27]. The World Health Organization (WHO) claims that all cases of bloody diarrhea should be treated with antibiotics. Effective antibiotic therapy lowers the risk of serious complications and death, shortens symptom duration, and decreases the shedding of organisms in stools [16, 31]. In our case, *S. flexneri* was resistant to ampicillin, cefotaxime, ceftriaxone, cefixime, and cotrimoxazole. There are different mechanisms by which *Shigella* spp. can develop resistance to the therapeutic antibiotics, including production of enzymes (such as β-lactamases), cellular impermeability, alternate metabolic pathways, extrusion of drugs by efflux pumps overexpression, and drug treatment mutation [32, 33]. As a result, the initial empirical treatment with ceftriaxone was unsuccessful. However, a subsequent change in the treatment regimen to gentamicin and ciprofloxacin, to which the isolate was susceptible, resulted in a successful clinical outcome and complete resolution of the infection. Jain et al., from Delhi, reported a case of *S. flexneri* septicemia in an infant that was also resistant to ceftriaxone, resulting in the patient’s death [19]. In contrast, some studies indicated that ceftriaxone remains an appropriate option for treating *S. flexneri* bloodstream infection [7, 17, 18].

Along with antibiotic resistance, other risk factors, rather than antibiotic resistance, should also be considered in the development of *Shigella* bacteremia and its associated mortality. Few studies have addressed these risks. However, host factors such as age, malnutrition, non-breastfeeding, and immunocompromised status have been linked to death from *Shigella* bacteremia [34, 35].

In conclusion, although *S. flexneri* bacteremia is rare, it occurs mainly in children under five years of age. Therefore, blood and stool cultures should be taken for better management and prognosis in bloody gastroenteritis with fever in children. An early and accurate diagnosis is crucial because appropriate antibiotics and supportive care can be lifesaving for such patients. This study was the first report of *Shigella flexneri* serotype 1c isolated from blood in Iran, which possessed all investigated virulence factors. However, it remains uncertain whether this serotype possesses increased virulence. This rare case highlights the need for clinicians and microbiologists to characterize the role of these genes and other related virulence factors of *Shigella* spp. for successfully spreading this pathogen into blood circulation.

## Data Availability

Not applicable.
